# The relationship between isolated pes equinovarus and aneuploidies and perinatal outcomes: Results of a tertiary center

**DOI:** 10.4274/tjod.galenos.2020.60669

**Published:** 2020-12-10

**Authors:** Mete Sucu, Süleyman Cansun Demir

**Affiliations:** 1 Çukurova University Faculty of Medicine, Department of Obstetrics and Gynecology, Adana, Turkey

**Keywords:** Clubfoot, Down syndrome, diagnostic imaging, karyotyping

## Abstract

**Objective:**

Congenital pes equinovarus (PEV) is the most common congenital deformity of the foot, characterized by plantar flexion with a frequency of 0.2-0.3%. It can be diagnosed from the 12^th^ week of pregnancy. Non-isolated cases tend to be syndromic and complex. We aimed to evaluate the results of perinatally diagnosed isolated PEV.

**Materials and Methods:**

This was a retrospective cohort study conducted between March 2015-March 2020. Women who presented for fetal anomaly screening or were referred due to any suspected fetal anomaly were subjected to detailed fetal anomaly scans and checked for the presence of PEV. Karyotype analysis was discussed for patients with PEV. Pregnancy termination was recommended for those with chromosomal/life-threatening anomalies. The diagnosis was confirmed by postnatal examination/autopsy. Postnatal diagnosis was accepted as false-positive in those with no PEV.

**Results:**

One-hundred thirty-eight patients were found to have PEV, 41 (29.7%) of which were isolated. In the isolated group, the false-positive rate in the first trimester was significantly higher compared with the second trimester, 50%/15.3%, respectively (p<0.05). Chromosomal anomalies were detected in 2 (4.8%) patients in the isolated group. Termination was performed to 1 (2.4%) patients due to trisomy 21. In the non-isolated group, chromosomal anomalies were detected in 13 (13.4%) patients, and termination was recommended. Termination was also recommended to 18 (18.5%) patients due to anomalies incompatible with life. In the postnatal evaluation, the surgical treatment rate in the isolated/non-isolated groups was 6%/39.7% (p<0.05).

**Conclusion:**

When PEV is diagnosed, detailed fetal anomaly screening must be performed, patients should be informed about the chromosomal anomaly risk. High false-positive rates in the first trimester should be kept in mind for diagnosis. Karyotype analysis should be recommended also to isolated cases. It should be remembered that some neuromuscular/skeletal system anomalies may occur for the first time in the postnatal period in isolated cases.

**PRECIS:** We aimed to evaluate the perinatal and neonatal results of pregnant women who were found to have isolated PEV during the first and second trimester ultrasonographic screening in our clinic.

## Introduction

Congenital pes equinovarus (PEV) or clubfoot is a congenital malformation characterized by excessive plantar flexion of one foot or both feet, inward tilting of the heel, and adduction of the forefoot^(1)^. It is seen with a frequency of 2-3 per 1000 live births and is the most common congenital deformity of the foot^(2)^. It can be unilateral or bilateral, and its incidence in male fetuses is 2 times higher^(3)^.

**Figure 1:**
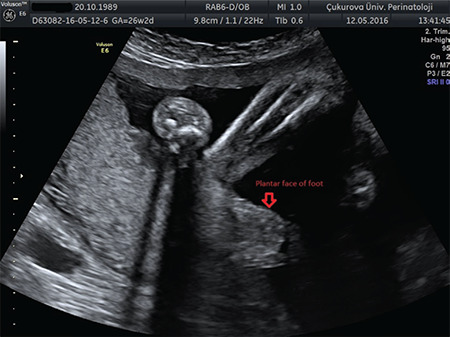
PEV ultrasonography image PEV: Pes equinovarus

Although the diagnosis of congenital PEV is usually made from the second trimester with ultrasonography (Figure 1), it is possible to diagnose it from 12^th^ week in the late first trimester^(4)^. Congenital PEV can be diagnosed more frequently through the development of ultrasound technology and the increase in the skills of physicians over time.

Syndromic cases tend to be more complex, and their association with other congenital malformations and/or chromosomal and genetic anomalies is common. It has been associated with some aneuploidies, deletion syndromes, sex chromosome abnormalities, neuromuscular disorders, microdeletion and duplications. Despite advances in molecular gene studies, a major causative gene has not been identified and clinical features of even familial cases show heterogeneity^(5)^.

Positional PEV is mostly associated with intrauterine factors limiting fetal movements, such as oligohydramnios, twin pregnancy and uterine anomalies. Early amniocentesis is one of the iatrogenic reasons that may lead to this^(6)^. There may be accompanying anomalies or it can be seen as isolated. Idiopathic PEV is usually isolated and generally have a good prognosis, their relationship with chromosomal anomalies is limited, and familial cases have been reported^(5)^. The fact that it has a higher prevalence in some populations and that it is more common among the male sex suggests that it is the result of a polygenetic predisposition^(4)^.

Environmental and genetic factors are thought to play a role in the etiology. It has been shown that maternal smoking, increased body mass index (BMI) and selective serotonin reuptake inhibitor use in the first trimester increases the risk of congenital PEV^(7,8,9)^. It was reported that Lamotrigine exposure during the prenatal period increased the risk of congenital PEV^(10)^.

Conservative treatment is the primary method recommended in PEV treatment. Surgery is recommended for patients who do not respond to conservative treatment or who are late for treatment. Untreated patients may experience limitation of motion, deformity, and pain in the long term. Conservative methods include corrective splints (Ponseti or Kite’s method), physiotherapy, and serial manipulations^(11,12)^. The surgical method may vary depending on the severity of the clinical course and accompanying anomalies^(13)^.

In this study, we aimed to evaluate the perinatal, neonatal and orthopedic results of pregnant women who were found to have isolated PEV during the first and second-trimester ultrasonographic screening in our clinic.

## Materials and Methods

This study was a retrospective cohort study conducted in the Perinatology Unit of Çukurova University Faculty of Medicine, Department of Obstetrics and Gynecology, between March 2015 and March 2020. Patients who presented to our clinic for routine first-trimester and second-trimester fetal anomaly screening and patients referred to our clinic due to any suspected fetal anomaly or positive screening test were included in this study. Among the patients admitted in the first trimester, those between 12 and 14 weeks were included in the study. All patients were subjected to a detailed fetal anomaly scan and checked for the presence of PEV. Sonographic examinations of all patients were performed by four researchers experienced in fetal anomaly screening using a transabdominal 4-8-MHz probe with a Voluson E6 (GE Medical Systems, Zipf, Austria) device, and information of all patients was recorded on the Microsoft Viewpoint (GE Medical Systems) data recording system. Information of the patients and accompanying anomalies were obtained retrospectively from patient files and Microsoft Viewpoint (GE Medical Systems) program. This study comprised a heterogeneous patient group including high and low-risk patients. Approval for the study was obtained from the Ethics Committee of Çukurova University Medical Faculty (Registration number: 02.02.2018-74-1). Written informed consent was obtained from all patients.

When PEV was detected, fetal karyotyping was recommended to all patients by performing amniocentesis. Termination of pregnancy was recommended for patients that were detected to have karyotype anomalies and/or severe life-threatening anomalies. Patients whose pregnancy was terminated were not included in the postnatal evaluation. All patients were examined by a pediatrician and an orthopedist after birth. Patients who had no PEV in the neonatal examination were considered as false-positive and they were excluded from the postnatal evaluation. In patients with suspected findings of chromosomal anomalies in postnatal examinations, this was confirmed by karyotype analysis in peripheral blood. In the absence of suspected findings, the karyotype was accepted as normal.

### Statistical Analysis

All information including the gestational week at the time of diagnosis, maternal age, BMI, accompanying anomalies, karyotype results, birth week, mode of delivery, and post-natal results were recorded and analyzed. Statistical analysis was performed using the Microsoft SPSS Excel program. The chi-square test was used for comparisons. P-values of <0.05 were considered statistically significant.

## Results

During the study period, 7680 patients who presented for routine first and second-trimester fetal ultrasonography screening or who were referred to our clinic due to any suspected fetal anomalies or positive screening tests were examined. One hundred thirty-eight patients with congenital PEV were included in the study. In 41 (29.7%) patients, no accompanying fetal anomalies were observed. In 97 (70.3%) patients, there were some additional fetal anomalies together with PEV. The majority of the patients were diagnosed in the second trimester [128/138 (92.7%)]. Patients diagnosed in the late first trimester (12-13 weeks and 6 days) constituted 7.3% (10/138). Sixty-four (65.9%) patients in the non-isolated group agreed to undergo amniocentesis and chromosomal anomalies were detected in 13 patients. Termination of pregnancy was recommended for 31 patients in the non-isolated group due to severe anomalies. The distribution of these severe anomalies in the non-isolated group was as follows: trisomy 18 in nine patients, trisomy 21 in three patients, trisomy 13 in one patient, neural tube defect in 11 patients, lethal skeletal dysplasia in four patients, and major cardiac anomalies in three patients. Of the 31 patients for whom termination was recommended, 29 accepted the termination. The pregnancy of the remaining 68 patients was followed-up until delivery. Nineteen (46.3%) patients in the isolated group agreed to undergo amniocentesis and chromosomal anomalies were detected in two patients (trisomy 21 in one patient, 47 XXY in one patient). Pregnancy termination was performed to one (2.4%) patient in the isolated group due to trisomy 21.

**Figure 2:**
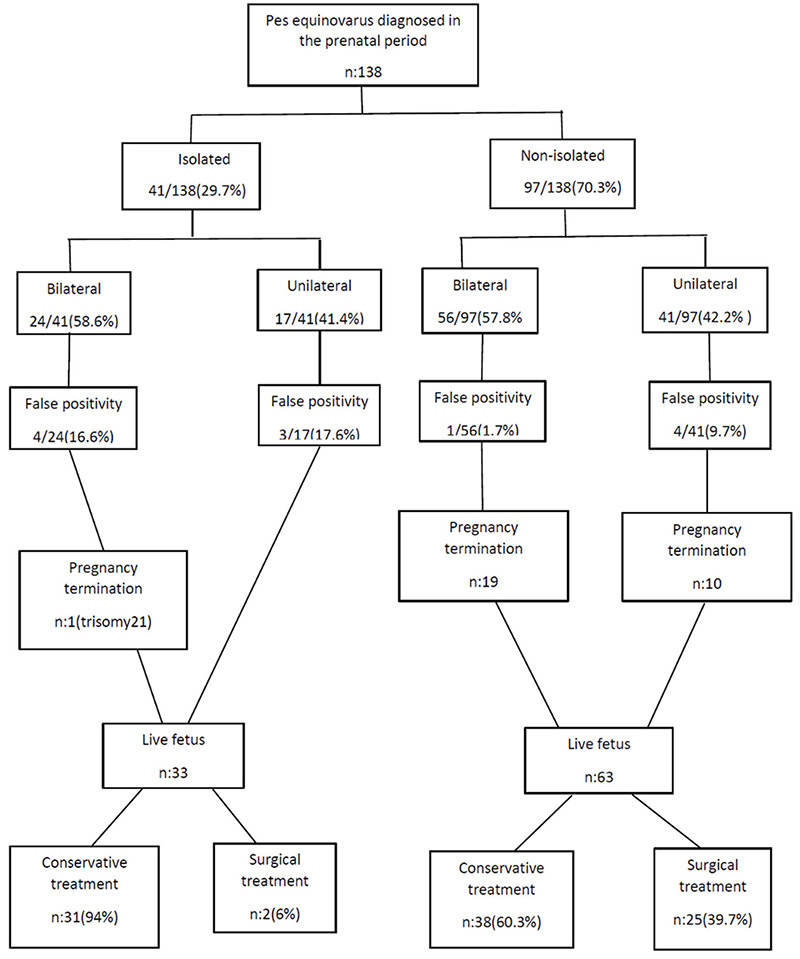
Clinical course of PEV cases diagnosed in the prenatal period PEV: Pes equinovarus

PEV was unilateral in 58 (42%) patients and bilateral in 80 (58%) patients. The rate of unilaterality was 41.4% (17/41) in the isolated group and was 42.2% (41/97) in the non-isolated group, the difference was not statistically significant (p<0.05). The clinical course of patients diagnosed as having PEV in the prenatal period is summarized in Figure 2.

In the postpartum evaluations (examination and autopsy) performed to confirm the diagnosis of PEV, it was found that some fetuses did not have PEV. When the isolated group and the non-isolated group were compared for false positivity, the false-positive rate was significantly higher in the isolated group (Table 1). The rate of false positivity in the isolated group was found as 17% (7/41). When the isolated group was divided into unilateral and bilateral subgroups and examined, false-positive rates were similar. In the non-isolated group, the rate of false-positives was 5.1% (5/97), and the rate in the unilateral and bilateral subgroups was 9.7% (4/41) and 1.7% (1/56), respectively. The difference between the percentages was not statistically significant (p>0.05).

When the false-positive rates were evaluated according to the week of gestation at the time of diagnosis, the false-positive rates were detected to be significantly higher in the first trimester (Table 2). False positivity was detected in three (30%) of 10 patients diagnosed as having PEV in the first trimester, and nine (7%) of 128 patients diagnosed at the second trimester were found to be false positives. The false-positive rate in the isolated group was up to 50% in the first trimester (Table 2).

When the sex distribution was examined after removing the false-positive cases, the percentage of male fetuses in the isolated and non-isolated groups was 64.7% (22/34) and 66.3% (61/92), respectively, similar to each other and almost twice higher than that of female fetuses.

When compared for characteristic features, no significant difference was found between isolated and non-isolated groups in terms of age, BMI, conception type, unilaterality/bilaterality, mode of delivery, and sex distribution. The clinical features of patients with PEV diagnosed during the prenatal period are shown in Table 3. The mean gestational week at the time of diagnosis, the mean birth week, and mean birth weight was significantly lower in the non-isolated group (Table 3). The rate of pregnancy termination was 2.9% in the isolated group, whereas it was 31.5% in the non-isolated group, the difference was statistically significant (p<0.05). The rate of need for intensive care in the neonatal period was 10% in the isolated group, whereas it was 44% in the non-isolated group, the difference was statistically significant (p<0.05). In the neonatal examination, suspicious findings for chromosomal anomalies were found in two patients in the non-isolated group, and they were confirmed by karyotype analysis in peripheral blood. Trisomy 21 was detected in one patient and 47 XXY in one patient.

When the frequency of chromosomal anomalies was evaluated according to unilaterality or bilaterality, it was found as 5.88% (3/51) in the unilateral group and 20% (15/75) in the bilateral group, the difference was statistically significant (p<0.05).

Trisomy 21 was detected in 51 patients in the entire study group [0.66% (51/7680)]. In five of 51 fetuses with trisomy 21, 9.8% (5/51) had PEV. After the elimination of patients with trisomy 21 in the entire group, the PEV rate was calculated as 1.58% (121/7639). The rate of trisomy 21 in patients with PEV was 3.96% (5/126). However, the rate of trisomy 21 in the group without PEV was found as 0.6% (46/7554). This indicates a 6.6-fold increase in the risk for trisomy 21 in patients with PEV.

Thirty-one (94%) patients in the isolated PEV group responded to conservative treatment. Conservative treatment was performed on 38 (60.3%) patients in the non-isolated group; the difference was statistically significant (p<0.05). The conservative treatment rate was statistically significantly higher in the isolated group (p<0.05). Surgical treatment was performed on patients who did not respond to conservative treatment or who were late for treatment. Corrective casting (Ponseti) and physiotherapy were used in conservative treatment. In terms of surgery, the most preferred methods were achillotomy and posteromedial release surgery.

## Discussion

First-trimester and second-trimester fetal anomaly screening are recommended to all pregnant women during antenatal follow-up. Although PEV incidence is stated as 0.2-0.3% in the literature, the results in our study were far from these values, as some of the patients were referred. Although the diagnosis of PEV is usually made in the second trimester, Keret et al.^(4)^ showed that it was possible to make a diagnosis from the 12^th^ week in the first trimester. However, the diagnosis made in the first trimester has some drawbacks. Bogers et al.^(14)^ showed that there was a temporary PEV position during the normal development of the lower extremity in the first trimester and this development continued until the 13^th^ week. Diagnoses made without waiting for this physiologic positional change will cause an increase in false positivity. In our study, the rate of false positivity in the entire group was 12/138 (8.69%). The false-positive rate was statistically significantly higher in patients in the first trimester than in the second trimester (30% vs. 7%) (p<0.05). In the isolated group, the false-positive rate was found as 7/41 (17%), reaching 50% in the first trimester. In the literature, this rate varies between 0% and 40% for isolated cases^(15,16,17,18)^. Our findings were similar to the literature. However, in the isolated group, 50% false positivity in patients diagnosed in the first trimester draws attention, which can be explained by the fact that the patients in the isolated group were milder and by the physiologic position change of the foot in the first trimester. Due to high false-positive rates, isolated cases, especially those diagnosed in the first trimester, should be followed up with serial examinations to confirm the diagnosis.

In this study, the median gestational week at the time of diagnosis of PEV was determined as 21.5 and 19.1 for the isolated and non-isolated groups, respectively. This can be explained by the fact that the disease is more complex and severe in the presence of accompanying anomalies and therefore can be diagnosed earlier^(15,19,20)^. Hartge et al.^(21)^ reported the median gestational week at the time of diagnosis as 23 weeks.

Postnatal examinations allow the detection of accompanying findings that could not be found in the prenatal period. Hence, in the non-isolated group, chromosomal anomalies were found in two patients who did not undergo amniocentesis during the antenatal period (trisomy 21 in one patient, 47 XXY in one patient). In the isolated group, no additional anomalies and chromosomal anomalies were found in the postnatal period. Lauson et al.^(22)^ stated that neurologic, developmental, and additional structural anomalies could be detected in postnatal follow-up in isolated cases. In their studies, it was observed that 10% of isolated cases turned into complex cases after a minimum of 1-year follow-up. Di Mascio et al.^(20)^ reported that anomalies related to the skeletal system and neuromuscular system were detected at a rate of 7% in the postnatal follow-up of patients diagnosed as having isolated PEV in the prenatal period. Offerdal et al.^(15)^ made a minimum of 2-years follow-up and found that 15% of the cases turned into complex cases. Shipp and Benacerraf^(16)^ found higher rates in their study. Our study has limitations in this regard because we have not yet followed-up all patients for at least one year.

Our sex distribution (boy/girl) in isolated PEV cases was 2:1, which was consistent with the literature. In our study, the percentage of non-isolated PEV was 73%. Although this rate varies between 48% and 51% in some community studies, rates up to 80% have been reported in tertiary centers such as our clinic where high-risk patients are treated^(15,17,23)^.

In our study, although chromosome anomalies were detected with a rate of 5.8% in the isolated group, the rate was 16.3% in the non-isolated group. Many authors believe that karyotype is necessary in the presence of anomalies accompanying PEV; however, there are ongoing discussions for isolated cases^(24,25)^. The most common chromosomal anomalies associated with PEV are trisomies 13 and 18. It is possible to recognize cases with trisomy 13 and 18 using ultrasonography due to accompanying anomalies. However, sex chromosome anomalies and trisomy 21 sometimes display very limited or no findings. For this reason, especially in isolated PEV cases, karyotyping comes to the fore due to the risk of trisomy 21. In the study of Viaris et al.^(26)^, the rate of chromosomal anomalies in the isolated group was 2.2%. Lauson et al.^(22)^ reported the incidence of chromosomal anomalies as 2.3% in the isolated group. Recommending karyotype to isolated cases in the presence of advanced maternal age or positive screening test should not be avoided^(20)^. PEV was detected in five of 51 patients with trisomy 21 in our study population (9.8%). This situation increases the risk of trisomy 21 in patients with PEV 6.6 times. Offerdal et al.^(15)^detected aneuploidy at the rate of 13% in patients with PEV and showed that there was a strong relationship between PEV and chromosomal anomalies, and they recommended karyotyping for all patients including isolated cases. Tegnanader and Eik-Nes^(27)^ reached similar results.

According to the findings of Bakalis et al.^(17)^, the prognosis was worse and the frequency of chromosomal anomalies was higher in the bilateral group, similar to our study. In the study of Viaris et al.^(26)^, no difference was found between the two groups.

Despite the high incidence of PEV, only a few causative genes are known. *PITX1 (MIM 602149), IGFBP3 (MIM 146732), TBX4*, and *RBM10* genes have been found to be associated with isolated PEV^(28,29,30)^. Recommending chromosomal microarray studies in addition to conventional karyotyping to patients with PEV will help to better understand the factors causing the disease because multigenetic factors play a role in the etiology^(20)^.

Conservative treatment is the primary method recommended in the treatment of PEV. Surgery is recommended for patients who do not respond to conservative treatment. There is no treatment in the prenatal period. The accepted method in conservative treatment is Ponseti casting (12). In our cases, surgical treatment was performed on two patients (6%) in the isolated group and 25 (39.8%) patients in the non-isolated group, the difference was statistically significant (p<0.05). Careful fetal anomaly screening performed to detect accompanying anomalies will help us understand whether the cases are isolated and predict the postnatal prognosis.

### Study Limitations

The limitations of this study are that it had a retrospective design, only patients diagnosed during the intrauterine period could be reached, and the rate of PEV detection could not be specified due to the exclusion of patients diagnosed for the first time in the postnatal period.

Another limitation is that only conventional karyotyping could be performed in patients, chromosomal microarray was not performed. Studies involving chromosomal microarray evaluations are needed to better understand the etiology of the disease.

## Conclusion

The diagnosis of PEV can be made in the late first and second trimesters. In the first trimester, the rate of false positivity is higher, and the diagnosis should be confirmed with serial examinations. When PEV is diagnosed, detailed fetal anomaly screening should be performed for anomalies that may accompany, and patients should be informed about the increased incidence of chromosomal anomalies, and karyotype and chromosomal microarray analysis should be recommended. Chromosomal microarray can identify clinically significant chromosome abnormalities (gains and losses of DNA) that are below the resolution of conventional chromosome analysis. The risk of this chromosomal and structural anomaly further increases in the presence of accompanying additional findings. It should be kept in mind that some neuromuscular and skeletal system anomalies may occur for the first time in the postnatal period in isolated cases.

## Figures and Tables

**Table 1:**
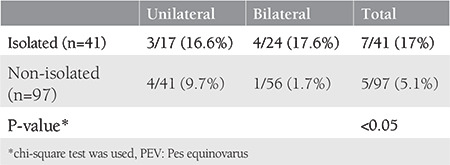
Distribution of postnatal false positivity rate of PEV due to laterality in antenatally diagnosed isolated and non-isolated groups

**Table 2:**
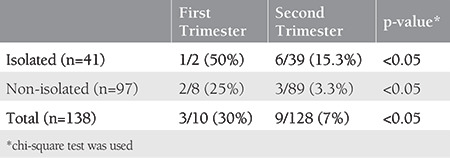
Postnatal false positivity distribution according to the gestational week at the time of antenatal diagnosis in the isolated and non-isolated groups

**Table 3:**
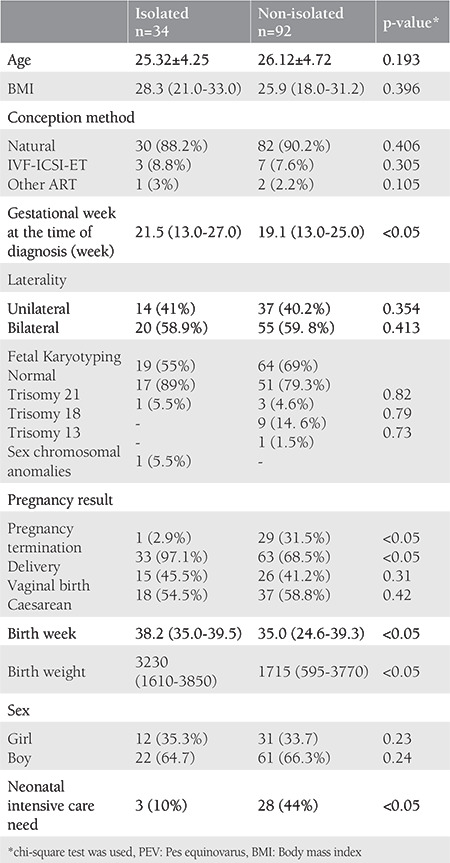
Clinical features of PEV patients diagnosed in the prenatal period
